# Immunologic Monitoring of T-Lymphocyte Subsets and HLA-DR-Positive Monocytes in Kidney Transplant Recipients: A Prospective, Observational Cohort Study: Erratum

**DOI:** 10.1097/01.md.0000479946.86789.21

**Published:** 2016-01-22

In the article “Immunologic Monitoring of T-Lymphocyte Subsets and HLA-DR-Positive Monocytes in Kidney Transplant Recipients: A Prospective, Observational Cohort Study”,^1^ which appeared in Volume 94, Issue 44 of *Medicine*, several typographical errors were reported by the authors. The article has since been corrected online.
